# Parent Perspectives of an Occupational Therapy Telehealth Intervention

**DOI:** 10.5195/ijt.2019.6274

**Published:** 2019-06-12

**Authors:** ANNA WALLISCH, LAUREN LITTLE, ELLEN POPE, WINNIE DUNN

**Affiliations:** 1 JUNIPER GARDENS CHILDREN’S PROJECT, UNIVERSITY OF KANSAS, KANSAS CITY, KS, USA; 2 OCCUPATIONAL THERAPY, RUSH UNIVERSITY, CHICAGO, IL, USA; 3 DUNN AND POPE COACHING, SANTA FE, NM, USA; 4 OCCUPATIONAL THERAPY, UNIVERSITY OF MISSOURI, COLUMBIA, MO, USA

**Keywords:** Autism Spectrum Disorder, Coaching, Occupational Therapy

## Abstract

Occupational therapy services delivered via telehealth can support families of young children with autism spectrum disorders (ASD) in everyday routines such as mealtime, bedtime, and play. The aim of the current study was to understand the lived experiences of parents who participated in a 12-week, telehealth-delivered occupational therapy intervention (Occupation-Based Coaching). We used semi-structured interviews and subsequent thematic content analysis to understand how parents perceived the mechanism of service delivery (i.e., videoconferencing) and the content of the intervention. Themes that emerged from the data included *Compatibility with Everyday Life*, *Collaborative Relationship*, and *Parent Empowerment*. Parents expressed how telehealth fit within their daily lives, how telehealth supported a collaborative relationship with the occupational therapist, and how the content of the intervention built a sense of empowerment.

Research has established that early intervention services promote positive developmental trajectories for children with autism spectrum disorders (ASD) and their families (Dawson, 2008; Estes et al., 2014; McConachie, Randle, Hammal, & Le Couteur et al., 2005). However, some families face many difficulties when accessing intervention services and these burdens are often magnified for underserved families (i.e., rural, low socioeconomic status, racial/ethnic minorities). Additionally, when children age out of early intervention, school-based intervention services must address educationally relevant goals. During early childhood, then, parents often do not have support for increasing children’s participation in home-based routines such as toilet training, mealtime behavior, bath time, or bedtime. Telehealth is a promising method to deliver intervention services to an increased number of families of children with ASD and address the gaps in service delivery from early intervention to early childhood.

The current study draws from a larger study delivering occupational therapy services for families of children with ASD via telehealth (Little, Pope, Wallisch, & Dunn, 2018a). While the pilot study’s quantitative findings showed that an occupational therapy intervention (called Occupation-Based Coaching) delivered via telehealth significantly increased parent self-efficacy and child participation, the current study followed up with parents using a qualitative approach. By understanding the lived experiences of parents that used occupational therapy telehealth services, we may gain insight into how to better design and tailor telehealth interventions for specific needs of parents and children with ASD.

Telehealth is a mechanism that allows intervention services to be delivered at a distance, and research is pointing to the effectiveness of occupational therapy delivered via telehealth (e.g., American Occupational Therapy Association, 2018; Cason, 2011; Little et al., 2018a; Zylstra, 2013). When occupational therapy interventions for young children with ASD utilize innovative methods of service delivery, we may increase the number of families that receive services to positively influence child and family outcomes. However, more research is needed to elucidate the ways in which telehealth can best support families and by using a qualitative approach, we can understand how to better design interventions delivered via telehealth.

The Division of Early Childhood (DEC) recognizes coaching and direct collaboration with parents and caregivers as a best practice in early intervention (Division for Early Childhood, 2014), and the telehealth delivered occupational therapy program was based in these principles. Occupation Based Coaching (OBC) directly involves parents in creating goals and strategies to increase child participation in everyday contexts (see Little et al., 2018a for full description of intervention). OBC focuses on family-identified goals and capitalizes on family strengths to support child participation. During OBC, the occupational therapist uses reflective questioning and commenting to problem solve through potential strategies. This process allows parents to gain a deeper understanding of possible solutions, and ways to implement and evaluate the effectiveness of these solutions (Dunn, Little, Pope, & Wallisch, 2017; Little et al., 2018a; Rush & Shelden, 2011). Research suggests that OBC significantly increases parent self-competence (Little et al., 2018a). In one qualitative investigation of coaching for young children with ASD, Foster and colleagues found that parents perceived the parent-therapist relationship supported parents’ feelings of self-efficacy. Additionally, parents reported that the process of analyzing and reflecting with the therapist serving as the ‘coach’ was a core component of increasing self-efficacy (Foster, Dunn, Mische Lawson, 2013).

While the quantitative investigation of OBC delivered via telehealth showed significant increases in child participation and parent self-efficacy (Little et al., 2018a), it is vital to understand why and if parents’ perceptions of coaching as provided over videoconferencing are congruent with previous studies. Parent perspectives of the process of coaching may help in determining the active ingredients of this innovative intervention method. Therefore, this study explored the lived experiences of parents following participation in a 12-week telehealth intervention using OBC.

## METHODS

### PARTICIPANTS

This study used purposeful sampling, where participants in the intervention study were randomly recruited from a previous quantitative study. Participants were included in the larger study if parents had a child under 7 years of age and identified as having ASD per an interdisciplinary diagnostic team using gold standard measures (for full description see Little et al, 2018a). Additionally, all families needed to read/speak English fluently. For the current study, participants were excluded if they did not complete all post-intervention assessments. We randomly selected 9 participants out of 17, and 8 completed the interview. One participant indicated she was too busy to complete an interview at the time of data collection. Children’s ages in this sample ranged from 28–79 months (M= 50.13 months; SD= 15.09 months). All parents completed OBC sessions approximately once a week for 12 weeks and met with an OT for approximately 45–60 minutes. During the first session parents created goals with their therapist, and the goals for the 8 participants included: (1) mealtime, (2) toilet training, (3) social interactions, (4) communication, (5) sleep, (6) play, and (7) transitions. See Table 1 for participant characteristics.

### DESIGN

To understand parent perspectives of occupational therapy delivered via telehealth, we used semi-structured interviews. We then used a qualitative thematic content analysis approach described by Braun and Clarke (2006). Thematic content analysis is a method for identifying, analyzing and reporting data patterns or themes, to aid in organizing and providing rich data description. We used an inductive process to allow the codes and themes to emerge from the data. Inductive analysis derives data that is not driven by a researcher’s theoretical interest in the topic and minimizes the researcher’s analytic preconceptions (Braun & Clarke, 2006). This form of thematic analysis generates greater data driven codes, themes and ultimately results.

### PROCEDURES

This study was approved by the University of Kansas Institutional Review Board and all parents provided informed consent prior to starting the interview. Following semi-structured interview guides, two interviewers who were not previously familiar with the parents completed and recorded the interviews via Zoom. Participating parents were familiar with the Zoom videoconferencing technology since this was used in the larger pilot study and Zoom is a secure videoconferencing platform that offers end-to-end encryption and is compliant with the Health Insurance Portability Accountability Act of 1996 (Pub. L. 104-191). During the interview, researchers wrote field notes about emotions, intonation of voice during interview, and any apparent biases that arose during the interview (Creswell, Fetters, Piano Clark & Morales, 2009). Field notes aid in the overall credibility of the research study. Open-ended interview questions were adapted from a Foster, Dunn, and Lawson (2013) evaluation of an in-person coaching intervention and included:

1. What was different about your experience with telehealth versus other services?
2. What was the quality of your experience?
3. What did you like most about telehealth?
4. What did you like least or would like to change about telehealth?
5. What would you like to share with other parents about this experience?
6. Tell me some things that you understand differently after our experience together.
7. How was the telehealth intervention consistent with services you have used in the past?
8. Is there anything that you are doing differently after the telehealth intervention?
9. How do you problem-solve challenges now?
10. What was the most helpful component of the intervention process?

### DATA ANALYSIS

We transcribed interviews verbatim, and guided our analysis by the six steps outlined by Braun and Clarke (2006). These iterative steps included: (1) familiarizing yourself with the data, (2) generating initial codes, (3) searching for themes, (4) reviewing themes, (5) defining and naming themes, and (6) producing the report. Two authors reviewed the data to generate initial codes; then the two compared codes and came to agreement. The two authors then revisited the full transcriptions to ensure that the initial, agreed upon codes described the data. Using the codes, two of the authors then searched for themes and met multiple times to review the data as well as ensure that the names of the themes described the parent reports. Further, to provide a comprehensive report on the current study, we used the COnsolidated criteria for REporting Qualitative research (COREQ) 32 item checklist (Tong, Sainsbury, & Craig, 2007). This means we provided descriptive information on our research team (e.g., characteristics of interviewers and coders, relationship to research members to interviewees), participant selection (e.g., sampling, sample size, characteristics), setting (i.e., Zoom), data collection (e.g., interview guide, transcripts), data analysis (e.g., coders, codes, themes), and findings (e.g., quotations).

### FINDINGS

The findings revealed three themes which included: (1) Compatibility with Daily Life, (2) Collaborative Relationship, and (3) Parent Empowerment.

### COMPATIBILITY WITH DAILY LIFE

A major theme that emerged from parent reports about their experience with occupational therapy services via telehealth was *Compatibility with Daily Life*. This theme is reflected in parents’ discussions about how the structure of the telehealth service delivery model and the specific intervention model (OBC) fit within the structure of the family. This theme was prominent throughout the interviews with all parents. First, parents discussed how previous service delivery models were inconvenient or not meeting the needs of families, which we described as the subtheme: *Incongruent with Daily Life*. Parents discussed how the delivery and content of the intervention compared to other services that did not fit within their daily lives or did not meet parent goals. These statements indicated a difference in the OT’s perspective, as well as a difference in the goals other service delivery models addressed. For example, one mother stated:

“Her OT at school spent the entire school year working on fine motor stuff and okay, well it’s not a waste of time, but to me it's not the most important thing. And you know what [my daughter] didn't make any progress with her OT at school all year, there was no progress. And her OT in her IEP update said that there was no progress due to [my daughter’s] lack of interest. I'm [thinking], who's lack of interest are we referring to? I didn't like her approach, I liked [telehealth] in that I made my own goals and then we broke them down into steps that were really easy to implement.”

Another mother stated:

[I] personally prefer [the telehealth OBC intervention] because I can try these strategies at home. With occupational therapy in the school district, you never know what they’re working on. In outpatient, it’s great, but you were there to be more hands on. [With telehealth] we tried techniques together and I would report to [the OT] how they went.

Second, parents discussed the *Convenience* of the intervention, and how telehealth decreased travel and was more accessible for their family. One mother stated:

I have one and a half year old twins. It’s very difficult to go to offices and sit for an hour, get everybody out of the house and somewhere on time. So that was really convenient being able to do [telehealth] from home, and it only took an hour, it didn’t take an hour plus travel time.

Another mother expressed: “*What did I like the most? That we didn’t have to leave the house*.” Finally, one mother said “*I really liked that it was on our schedule.”*

Third, parents discussed the ways in which telehealth fit with their contexts, routines, and situations as a family; we labeled these parent reports as *Natural environment*. One mother expressed,

[The intervention] was very customized to our life and our routine and how we did things. It was awesome, instead of being like ‘here’s this technique make it work for you’. It was, ‘what did you do? Oh, maybe we can improve upon that. Let’s try a few different strategies.

Another mother reported: “*I just loved problem solving little issues we had throughout our week and how tailored or customized to our life it was.*” Telehealth, along with the content and process of the intervention fit within families’ natural environments.

### COLLABORATIVE RELATIONSHIP

In the telehealth OBC model, occupational therapists must trust that parents know what will work best for their child and that parents will implement intervention strategies that are compatible with their daily lives. The *Collaborative Relationship* theme echoed parents’ discussion of characteristics of an occupational therapist that supported a feeling of partnership throughout the intervention process. First, parents discussed the ways in which occupational therapy professional knowledge added to the online sessions. For example, one mother stated,

*She [the OT] influenced ideas…like my daughter doesn’t eat very well. Then [the OT] gave me the idea of well try [the food] so many times, then obviously she doesn’t like it. Well, okay, that makes sense. Cause I was beating myself up, like here eat a green bean, eat just one green bean. And you know what, I don’t eat green beans that great either*.

One mother said,

It can be helpful to have someone to talk to about challenges you have with your child, a professional who has some knowledge in the area and is able to give you positive praise as well as suggestions to help you problem solve.

In addition to the professional knowledge of the occupational therapist, the parents expressed that the ways in which the therapist was empathetic and did not judge parent decisions. One mother said *“I felt like my opinion or whatever I said [the OT] respected and valued my input.”* Another mother expressed:

I felt comfortable asking [the OT] questions, ‘what does this mean? and I didn’t feel judgment from her, you know telling me I’m doing something wrong…you kind of get that a lot. We’re always kind of nervous because we’re trying our best, you’re always hoping you’re doing it right…So I feel working together as a team was probably my favorite part.

Clearly, parents felt that the occupational therapist brought specific knowledge to the sessions, but was respectful of the decisions that parents made with regard to intervention strategies that they were going to try.

### PARENT EMPOWERMENT

Lastly, parents often discussed ways in which they felt more confident following the telehealth intervention. Parents discussed how they problem solved new situations; through the telehealth sessions, parents had the time to reflect on situations with the occupational therapist and gain confidence in trying new strategies. For example, one mother stated:

Normally I would’ve been fretting…instead I sat down with my child, and I asked him how he felt about [riding the bus]. And we talked about how he is nervous about riding the big bus, and we talked about how we could problem solve that…The OT gave me the confidence to sit down with my child and figure out what my child wanted…and truly he wanted to ride the big bus. He wanted to be with everybody else, which is awesome…Getting him more involved in his care, which I think as a parent, we take care of our children, but sometimes we don’t always involve them in the decisions, and for him that was a big deal.

Additionally, parents discussed their child’s strengths and felt they had a better understanding of their child’s behaviors. One mother stated:

Instead of immediately getting pissed off, I just try and breathe, and consider a lot more. What’s he going through? Why is he having a problem? Instead of just being annoyed that he’s not listening or he’s not doing what I’m asking him to do…and just saying, “Fine, I’ll do it” I’ll sit him on my lap and show him. [Telehealth] helped me to step back and take a deep breath and then look at it like, how can we learn from this, how can we do this different? How can we problem solve this?

Parents talked about a changed perspective about their child’s behaviors, and began discussing their child with greater positivity by mentioning child strengths, or their increase in patience with their child. One mother expressed: “*I’m more patient with him because I realize that he’s not doing anything wrong and we can problem solve and try different strategies to make something work.”* Another stated, *“I feel like I’m just thinking about our daily routine a lot more, and realizing that the things that we do and the techniques I use with my son- he can learn a lot more from them than I thought.”* Parents felt empowered to try new strategies, problem solve through situations, and consider their child’s strengths.

## DISCUSSION

The purpose of our study was to explore the lived experience of parents following a 12-week occupational therapy telehealth intervention. While interventions may show significant changes in parent and child factors, qualitative investigations are vital to elucidate active ingredients of treatment. Parent perceptions provided insight into how the occupational therapy model, OBC, delivered via telehealth was compatible with daily life, grounded in a collaborative parent-therapist relationship, and supported parents to feel empowered. These active ingredients may bolster current service delivery models and interventions to meet the needs of families of young children with ASD.

Parents indicated the intervention delivery method and content was highly convenient and compatible with their family’s daily life. These parent reports align with previous findings suggesting the convenience and usefulness of a telehealth platform (Ashburner, Vickerstaff, Beetge, & Copley, 2016; Benham & Gibbs, 2017; Cason, 2009; Pickard, Wainer, Bailey, & Ingersoll, 2016). For example, Pickard and colleagues (2016) provided a telehealth intervention to boost social communication skills in children with ASD, and parents described the convenience of the online sessions as well as described ways in which online sessions may have been beneficial for parents at the time of an ASD diagnosis. Further, Ashburner et al., (2016) found that parents reported that telehealth was more flexible than in-person services and could provide ongoing support. Others have suggested that when compared to other in-person delivery models, the convenience of telehealth models resulted in (1) fewer missed sessions, (2) a decrease in financial burdens on families and providers, and (3) better continuity of care (e.g., Covert, Slevin, & Hatterman, 2018; Lindgren et al., 2016; Little, Wallisch, Pope & Dunn, 2018b). Our current delivery models often require parents to drive for therapy appointments and this often poses a barrier to families, due to an increased time commitment and cost burden. Since many of the families included in the current study resided in rural areas and could not otherwise access home-based services, the reports of convenience may be amplified for the current sample. Additionally, while the intervention was convenient, technological difficulties (e.g., internet connectivity, software issues) may add barriers for families that are non-existent for in-person delivery models (Ashburner et al., 2016; Little, et al., 2018b). Overall, by providing services through platforms that are more compatible with the daily life of families, we may reduce many of the challenges associated with other service delivery models.

In a coaching model, parents’ goals and priorities guide intervention (Rush & Sheldon, 2011). Occupational therapists serve to support parents’ problem solving, reflection, and carry through with joint plans. The coaching model was evident in parent descriptions of the importance of ongoing collaboration with their occupational therapist. Throughout the interviews, parents reported how their opinions and perspectives drove the intervention, and they felt part of the “team.” In a telehealth model, it is clear that therapists must partner with parents. The model itself aims to build family capacity, and many families indicated the telehealth intervention allowed for them to be an active member of the intervention process; whereas, other models (e.g., school and outpatient services) were less transparent to parents. These findings are consistent with other studies suggesting family-centered care and parent-therapist relationships are active ingredients for interventions (e.g., Foster et al., 2013; Law et al., 2003). Overall, parents clearly described the benefits of jointly problem solving with an occupational therapist.

Building on family strengths and resources is recognized as a key component to family-centered practice (American Occupational Therapy Association, 2010; Division of Early Childhood, 2014). During OBC sessions in the current study therapists would often reflect on the strengths of the child, and problem solve with parents on strategies to build on these strengths. During the interviews parents often described their new perspective for focusing on what their child could do, rather than what they could not do. This is consistent with other studies suggesting that a focus on strengths may result in parents increasing positive statements, changing their perception of their child’s disability, and increasing their physical affection with their child (Carlson et al., 2010; Steiner, 2011). Given that a strengths-based approach is recognized as part of family-centered care, it is critical that parents in the current study recognized this as an active ingredient of OBC via telehealth and appeared to maintain this perspective upon study completion.

While there is a focus on tailoring specific interventions to child characteristics (e.g., The Interagency Autism Council Committee, 2012), emerging evidence suggests the efficacy of parent-implemented interventions is partially dependent on parent characteristics (Karst & Hecke, 2012; Osborne, McHuge, Saunders & Reed, 2008). Specifically, parent self-efficacy may predict parenting competence and child outcomes, and parents with increased self-efficacy demonstrate more effective strategies even when confronted with difficult child behaviors (Jones & Prinz, 2005). Our study gleans insight into how parents felt more empowered, or efficacious, following the OBC intervention. For example, many parents would indicate how they problem solve through difficult situations and their cognitive processes for determining the root of their child’s behavior. Our findings are consistent with qualitative reports of coaching reported in previous studies (Foster et al., 2013; Graham, Rodger, & Ziviani, 2014). For example, Graham et al., (2014) found that mothers’ perceptions of in-person coaching resulted in feelings of empowerment and increased time for reflection of how their own behaviors impacted child behaviors. This means the perceptions of parents experiencing OBC via telehealth were similar to parents experiencing in-person coaching models.

### LIMITATIONS

The sample of this study provides a wide range of perceptions from various income ranges and families living in rural areas; however, the small sample size and lack of racially and ethnically diverse participants reduces the generalizability of our findings. Thus it remains unclear if families who live within closer proximity to early intervention services would perceive the same active ingredients as seen in this study. Further, we added procedures to reduce potential bias (e.g., using an inductive process to analyze the data, parents completed the interview with unfamiliar research staff); however, the data analysis occurred with researchers who were familiar with the intervention. Since the researchers were familiar with the intervention and active ingredients, this may have led to biases when interpreting the findings. Lastly, many parents mentioned that telehealth was more convenient due to expanded hours (e.g., times outside of typical 8am-5pm business hours) and availability of the OT. We recognize that not all programs and occupational therapists may have the same flexibility in scheduling, even with the utilization of telehealth services. Overall, few studies have examined the parent perceptions of an OBC intervention via telehealth, and our findings provide critical knowledge to understand the complexities and meaningful components of an innovative service delivery model.

## Figures and Tables

**Table 1:** Participant Demographics (n=8)

Demographics	% (n)
Mother	87.5 (7)
Child gender (male)	62.5 (5)
Child race	
White	87.5 (7)
Multiracial	12.5 (1)
Child ethnicity	
Hispanic	12.5 (1)
Income	
<20,000	12.5 (1)
20,000–39,000	25.0 (2)
40,000–59,000	50.0 (4)
60,000–79,000	12.5 (1)
Mother’s education	
HS	0.0 (0)
Some college	62.5 (5)
Associates	12.5 (1)
Bachelor’s	0.0 (0)
Master’s	25.0 (2)
Father’s education	
HS	37.5 (3)
Some college	25,0 (2)
Associates	0.0 (0)
Bachelor’s	37.5 (3)
Master’s	0.0 (0)
